# Partial Egg Consumption Modifies the Diagnostic Performance of Allergy Tests to Predict Outcome of Double-Blind Placebo-Controlled Food Challenges to Egg

**DOI:** 10.1016/j.jaip.2023.12.036

**Published:** 2024-03

**Authors:** Andreina Marques-Mejias, Suzana Radulovic, Ru-Xin Foong, Irene Bartha, Marta Krawiec, Matthew Kwok, Zainab Jama, Faye Harrison, Cristian Ricci, Gideon Lack, George Du Toit, Alexandra F. Santos

**Affiliations:** a Department of Women and Children’s Health (Pediatric Allergy), School of Life Course Sciences, Faculty of Life Sciences and Medicine, King’s College London, London, UK; b Children’s Allergy Service, Evelina London Children’s Hospital, Guy’s and St. Thomas’ Hospital, London, UK; c Peter Gorer Department of Immunobiology, School of Immunology and Microbial Sciences, King’s College London, London, UK; d Africa Unit for Transdisciplinary Health Research (AUTHeR), North-West University, Potchefstroom, South Africa

**Keywords:** Baked egg, Basophil activation test, Egg allergy, Egg consumption, Food-frequency questionnaires

## Abstract

**Background:**

Many children are consuming some egg when they are diagnosed with egg allergy. We hypothesized that egg consumption could modify the diagnostic performance of allergy tests.

**Objective:**

To stratify diagnostic performance of tests according to egg consumption status.

**Methods:**

The BAT2 study (NCT03309488) participants underwent oral food challenge (OFC), food-frequency questionnaires, skin prick test (SPT), specific immunoglobulin E (sIgE) and specific immunoglobulin G4 (sIgG4) and basophil activation test (BAT).

**Results:**

At study entry, 45% of participants reported partial egg consumption (“consumers”) and 55% were avoiding egg strictly (“avoiders”). Avoiders had larger SPT (*P* < .001), higher BAT to egg (*P* < .001), sIgE to egg white (EW; *P* = .001) and to ovalbumin (OVA; *P* = .001), but not to ovomucoid (*P* = .231). Consumers had higher levels of sIgG4 to all egg allergens (*P* < .001) than avoiders. In consumers, the test with the best diagnostic performance was BAT (area under the curve [AUC] = .912) followed by SPT to raw egg (AUC = 0.805), EW-sIgE (AUC = 0.738), and OVA-sIgE (AUC = 0.732). In avoiders, the best tests were BAT (AUC = 0.834) and EW-sIgE (AUC = 0.833) followed by OVA-sIgE (AUC = 0.793) and SPT to EW (AUC=0.789). Using 100% sensitivity and 100% specificity cut-offs, the proportion of patients requiring OFC were 33% for BAT, 53% for SPT to raw egg, 61% for OVA-sIgE, and 73% for EW-sIgE for consumers; and 73% for BAT, 79% for EW-sIgE, and 93% for SPT to EW for avoiders.

**Conclusions:**

The diagnostic performance of tests is influenced by the immunomodulatory effect of egg consumption. BAT is the most reliable test and reduced the need for OFC, particularly in partial egg consumers.

***What is already known about this topic?*** Allergy tests, including skin prick test and specific immunoglobulin E, can support the diagnosis of immunoglobulin E–mediated egg allergy.

***What does this article add to our knowledge?*** Egg consumption can modify the predictive value of allergy tests, with the basophil activation test being most accurate in egg-partial consumers.

***How does this study impact current management guidelines?*** Assessing egg consumption when diagnosing egg allergy can inform the interpretation of test results and allow greater diagnostic precision.

## Introduction

Egg allergy is one of the most common food allergies in childhood and is outgrown in about 50% to 60% of children by 6 years of age.^1^^,^^2^ Over 80% of egg-allergic children tolerate some egg in the diet, often in the baked form, and this has been associated with a better prognosis.^3^, ^4^, ^5^ Furthermore, being able to eat baked egg (BE) has an undoubtedly positive impact on the quality of life of allergic children and their families by improving their nutrition and reducing restrictions to their social life.^5^ Previous studies have associated allergen-specific immunoglobulin E (IgE) levels and basophil activation with phenotypes of egg allergy^6^; however, there is currently no reliable objective marker to indicate when an egg-allergic patient is likely to tolerate BE.^7^, ^8^, ^9^, ^10^

In the BAT2 study (NCT03309488), we observed that many participants enrolled for assessment of possible egg allergy reported consumption of egg in the diet, which was often below the level of consumption appropriate for their age, and, surprisingly, some of these patients reacted during double-blind placebo-controlled food challenges (DBPCFCs) to BE. This observation motivated analyses looking at the relationship between pre–oral food challenge (OFC) egg consumption, test results, and OFC outcomes, to address this secondary end point of the study protocol. We hypothesized that consumption of egg could modify the results of skin prick test (SPT), basophil activation test (BAT), and allergen-specific immunoglobulin E (sIgE) and allergen-specific immunoglobulin G4 (sIgG4), and that this could be a reason why the results of tests currently available in the clinic were not good predictors of clinical reactivity to BE during OFC.

Herein, we report the egg consumption status in a cohort of children being assessed for egg allergy and its influence in the diagnostic performance of allergy tests to predict the outcome of DBPCFCs to BE.

## Methods

### BAT2 study participants

The BAT 2 study was a diagnostic study, designed according to the Standards for Reporting of Diagnostic Accuracy Studies (STARD) guidelines,^11^ in which children with suspected IgE-mediated allergy to either cow’s milk, egg, sesame, or cashew nut were prospectively recruited. Children assessed for possible egg allergy were submitted to DBPCFCs to BE and, if they passed this, to DBPCFCs to loosely cooked egg (LCE). On the day of the OFC, parents filled in a food-frequency questionnaire (FFQ) and the child had SPT and blood collection for serology and BAT. The study was approved by the London–Westminster Research Ethics Committee (reference 17/LO/0296) and the U.K. Health Research Authority. Informed consent was obtained from the adults with parental responsibility before any study procedures.

### Food-frequency questionnaires

We designed FFQs for egg which families filled in to report whether their child was consuming egg in the diet and the frequency and approximate amounts of BE, cooked egg as an ingredient, whole egg, or raw egg (RE), in relation to a typical age-appropriate portion size. FFQ were filled in at various study time-points: before and after the OFC to BE, after the OFC to LCE, and every 2 months for 2 years thereafter. We defined “consumers” as participants reporting the ingestion of any form of egg in the diet and “avoiders” as the participants who were avoiding all forms of egg strictly in the diet at study entry.

### SPT, sIgE and sIgG4

SPT were performed as previously described^12^ using a single-head metal lancet, 10 mg/mL histamine dihydrochloride as positive control; 50% glycerol and 50% buffered saline as negative control; egg white (EW) extract (ALK Abello, Madrid, Spain), RE (using fresh raw egg white), and BE (the latter using slurry made up of 1 g of the challenge food in 10 mL of saline). We aimed to compare the degree of IgE sensitization to the various egg preparations and the utility of SPT to RE, egg extract and BE (in decreasing order of allergenicity) in supporting the diagnosis of BE and LCE allergies. Skin weal diameter was recorded after 15 minutes by calculating the arithmetic average of 2 perpendicular diameters including the longest one.

Total IgE, sIgE, and IgG4 to egg, EW, ovalbumin (OVA), and ovomucoid (OVM) were determined using the standardized immunoenzymatic assay ImmunoCAP (Thermofisher, Uppsala, Sweden).

### Basophil activation test

The BAT was performed using heparinized venous blood on the same day of blood collection. Basophils in 100 μL of whole blood were stimulated with the same volume of egg extract (ALK-Abello), baked EW (Sigma-Aldrich, Poole, UK), anti-IgE (1 μg/mL, Sigma-Aldrich, Poole, United Kingdom) or *N*-formyl-methionyl-leucyl-phenylalanine (f-MLP; 1 μM, Sigma-Aldrich) diluted in Roswell Park Memorial Institute medium (GIBCO, Paisley, United Kingdom) with Roswell Park Memorial Institute alone as negative control. Cells were stained with CD123-FITC, CD203c-PE, human leukocyte antigen (HLA)-DR-PerCP, and CD63-APC (Biolegend, San Diego, Calif) and analyzed by flow cytometry using Fortessa with Diva software (BD Biosciences, San Jose, Calif). Data were analyzed with FlowJo software (version 7.6.1; TreeStar, Ashland, Ore) gating basophils as low side scatter/CD203c+/CD123+/HLA-DR-^11^^,^^17^ cells. Basophil activation was expressed as %CD63+ basophils.

### Oral food challenges

All study participants underwent DBPCFCs to BE, except infants who underwent open incremental OFC to BE. Individual doses were given according to the age of child, as indicated in Table E1 (available in this article’s Online Repository at www.jaci-inpractice.org). All negative DBPCFCs were followed by an age-appropriate open dose. If a reaction after a placebo dose occurred, a 2-day DBPCFC was performed. Participants who reacted to placebo on the 2-day DBPCFC, or who had a reaction following a placebo dose but refused to have a 2-day DBPCFC, or who did not complete or had an indeterminate OFC, were considered as not having an outcome and were excluded from the diagnostic analyses.

### Statistical analyses

Continuous variables were described using median and interquartile range. Consumers and avoiders were compared using the Mann-Whitney *U*-test. Categorial variables were reported by counts and percentages. Consumers and avoiders were compared by the χ^2^ or Fisher exact test when appropriate.

The receiver operating characteristic (ROC) curve analyses were the tools chosen to investigate the diagnostic accuracy of the various tests. The internal validity was investigated by bootstrap with 5,000 iterations. The Youden index was used to define the optimal cut-offs. The highest point in the ROC curve with 100% sensitivity and the lowest point in the same curve with 100% specificity defined positive and negative cut offs, respectively. Participants whose results fell within positive and negative cut-offs and for every sequence ended with OFC to clarify the allergic status of equivocal cases underwent sequential testing. All statistical evaluations were performed by SPSS v. 27. Statistical tests were 2-tailed and type-I error rate was set to 5% (α = 0.05).

According to our power calculation, a few cases in the range of 50 to 60 with an equal number of noncases would guarantee a statistical power in the range of 95% to 99% for a ROC curve having an area under the curve (AUC) of 0.75 or above, when considering a probability of false positive (type I error) in a range of 1% to 5%. The same number of cases and noncases and an AUC of 0.7 result in a statistical power in a range of 85% to 95%. The power calculation was conducted using the power.roc.test function from the pRoc package of the R software v. 4.0.2.

## Results

### Many children assessed for egg allergy reported partial egg consumption and such consumption was stopped after a positive challenge

**Figure 1:**
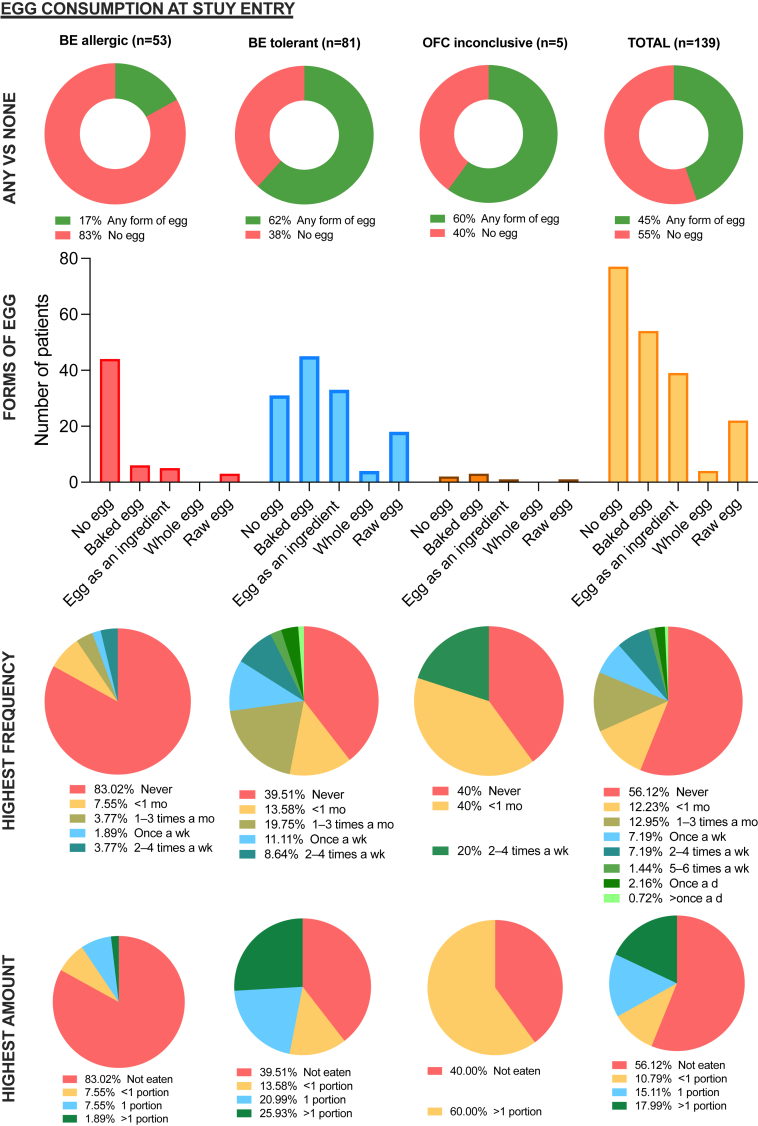
Egg consumption patterns at the time of the initial assessment and grouped by OFC outcome. Total, The whole study population; *BE Allergic,* baked egg allergic; *BE Tolerant,* baked egg tolerant; *Indet,* allergic status indeterminate due to inconclusive OFC.

**Figure E1:**
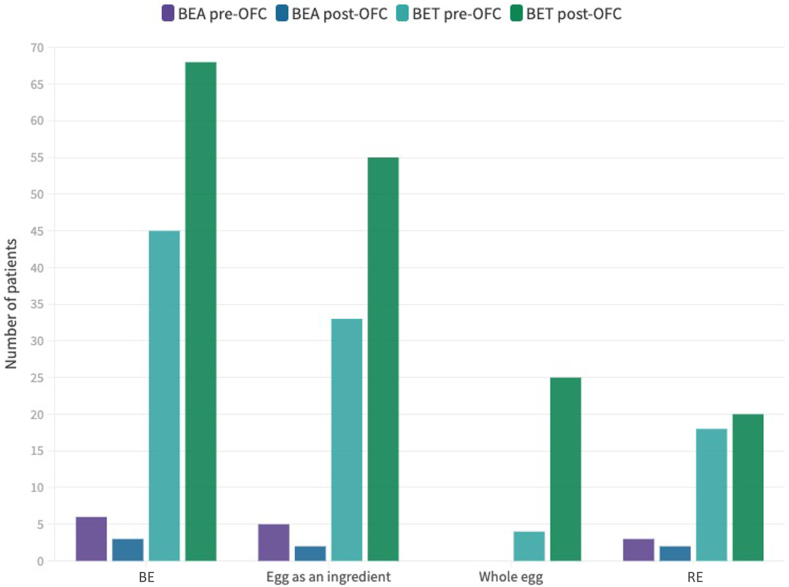
Pre– and post–baked egg (BE) oral food challenge (OFC) consumption of different forms of egg as reported in the food-frequency questionnaires (FFQs). *BEA,* Baked egg–allergic; *BET,* baked egg–tolerant; *RE,* raw egg.

Forty-five percent of participants reported egg consumption in some form and were defined as “consumers”; 55% were avoiding egg strictly and were defined as “avoiders.” Figure 1 illustrates the egg consumption in terms of egg forms, frequency, and amount in all participants and split by outcome of OFC in allergic, tolerant, and indeterminate-allergic status. Consumers were more likely to pass their DBPCFC than avoiders (62% and 17%, respectively; *P* < .001). After the OFC to BE, egg consumption decreased significantly in children who reacted or had an inconclusive OFC. As expected, BE consumption increased after a negative OFC (Figure E1; available in this article’s Online Repository at www.jaci-inpractice.org).

### Partial egg consumption is associated with differences in egg-specific immune response

**Figure 2:**
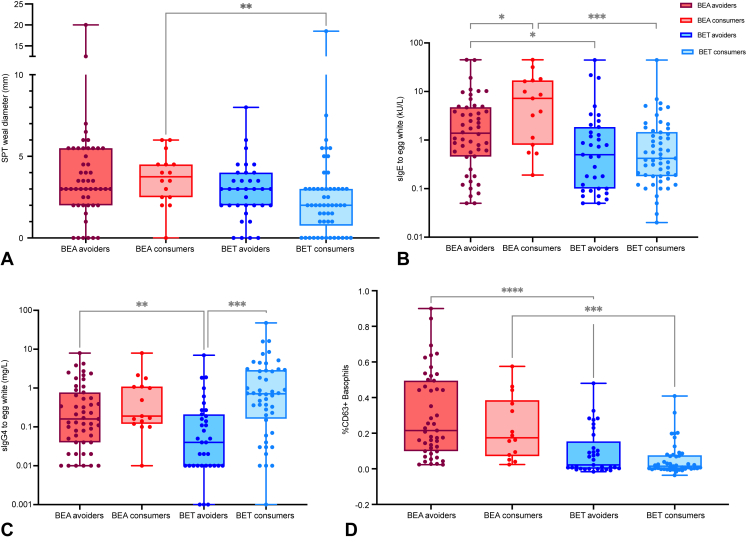
SPT to EW, sIgE to EW, sIgG4 to EW, and BAT following stimulation to egg extract in baked egg–allergic (BEA) avoiders and –consumers and baked egg–tolerant (BET) avoiders and –consumers. Statistically significant *P* values are indicated for comparisons made between BEA avoiders vs BEA consumers; BET avoiders vs BET consumers; BEA avoiders vs BET avoiders; BEA consumers vs BET consumers. ∗*P* < .05; ∗∗*P* < .01; ∗∗∗*P* < .001.

**Figure E2:**
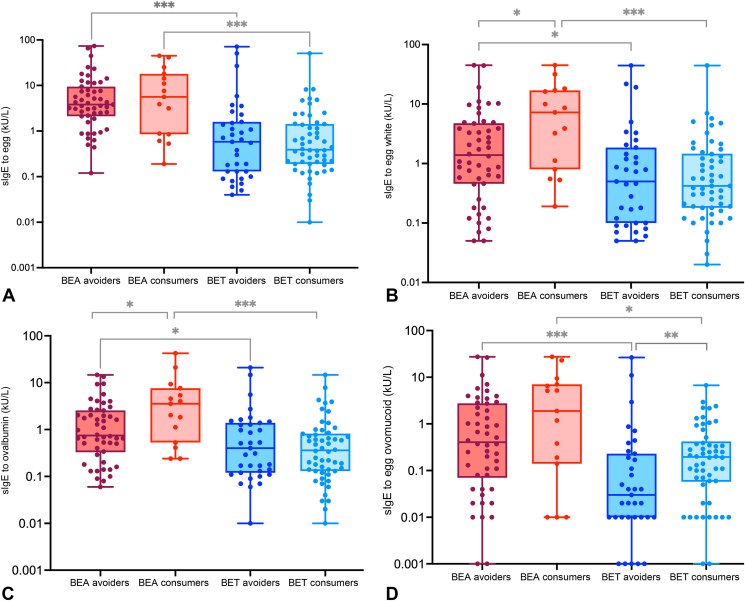
Specific immunoglobulin E (sIgE) to egg, egg white (EW), ovalbumin (OVA), and ovomucoid (OVM) in BEA avoiders and consumers and BET avoiders and consumers. ∗*P* < .05; ∗∗*P* < .01; ∗∗∗*P* < .001.

**Figure E3:**
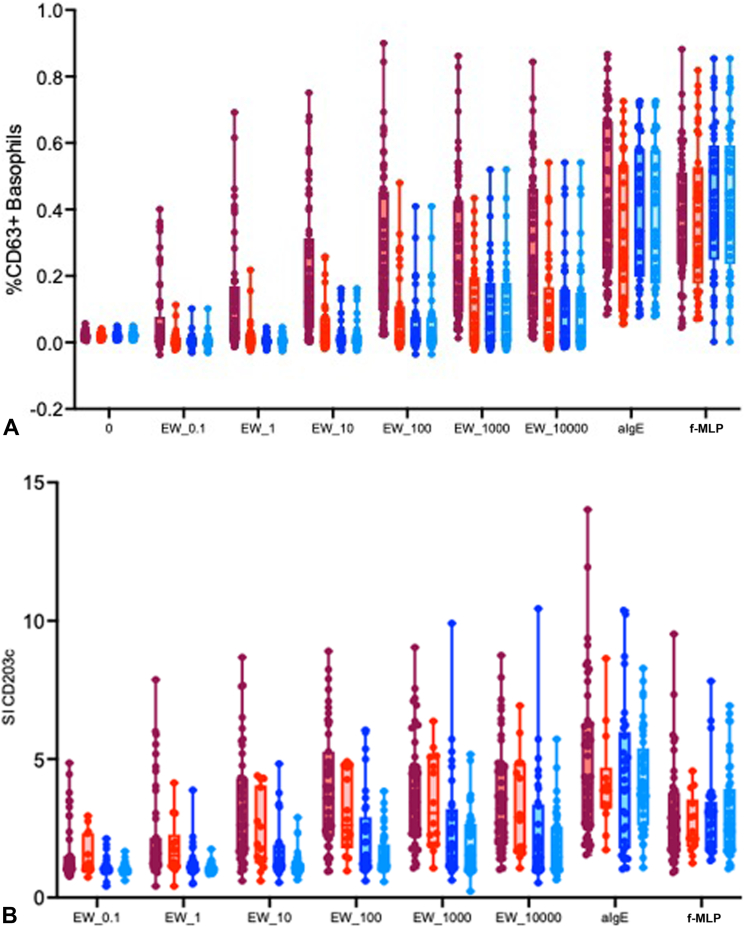
Basophil activation to EW (0.1–10,000 ng/mL) and controls (medium alone, anti-IgE (aIgE), and *N*-formyl-methionyl-flavin-5’-phenylalanine [f-MLP]) in BEA avoiders (dark red) and BEA consumers (orange) and BET avoiders (dark blue) and BET consumers (light blue) using (**A**) CD63 or (**B**) CD203c as activation markers.

**Figure E4:**
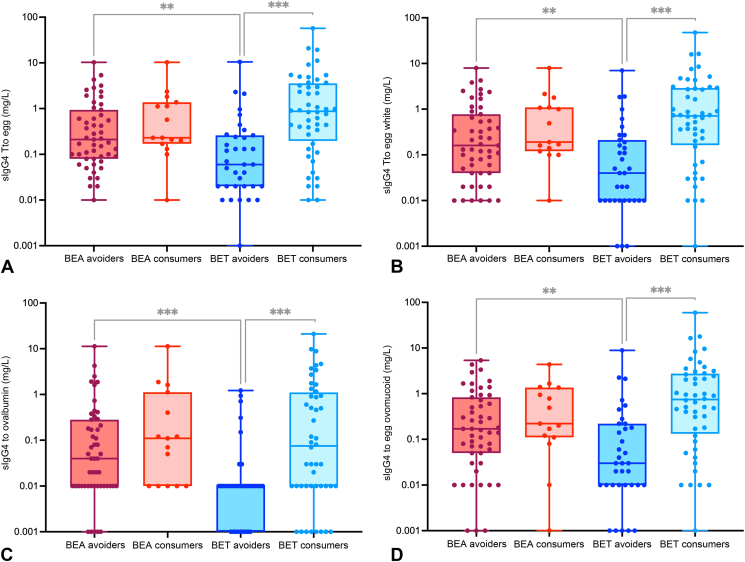
Specific immunoglobulin G4 (sIgG4) to egg, EW, OVA, and OVM in BEA avoiders and consumers and BET avoiders and consumers. ∗∗*P* < .01; ∗∗∗*P* < .001.

Table E2 (available in this article’s Online Repository at www.jaci-inpractice.org) represents the demographic and clinical characteristics of consumers and avoiders prior to OFC. Consumer and avoider groups did not significantly differ in sex, ethnicity, age, prevalence of eczema, allergic rhinitis, or asthma. Avoiders had significantly larger SPT to egg extracts, RE, and BE slurry (all *P* < .001); higher sIgE levels to egg (*P* = .002), to EW (*P* = .001) and to OVA (*P* = .001), but not to OVM (*P* = .231); and higher basophil activation following stimulation with various allergen concentrations (*P* < .001), but not non-allergen-specific controls. Consumers had higher levels of sIgG4 to egg, to EW, to OVA, and to OVM (all *P* < .001) than avoiders. Figure 2 and Figure E2, Figure E3, Figure E4 (available in this article’s Online Repository at www.jaci-inpractice.org) represent these differences graphically according to consumption and allergic status to BE and highlight the intergroup comparisons between allergic avoiders and allergic consumers, tolerant avoiders and tolerant consumers, allergic consumers and tolerant consumers, and allergic avoiders and tolerant avoiders. We also performed the 2 extreme comparisons—allergic consumers versus tolerant avoiders and allergic avoiders versus tolerant consumers—these comparisons were all statistically significant except for SPT to EW in BE-tolerant avoiders versus BE-allergic consumers; and sIgG4 to OVA between BE-tolerant consumers and BE-allergic avoiders.

### Diagnostic performance of tests differs with egg consumption status

**Figure 3:**
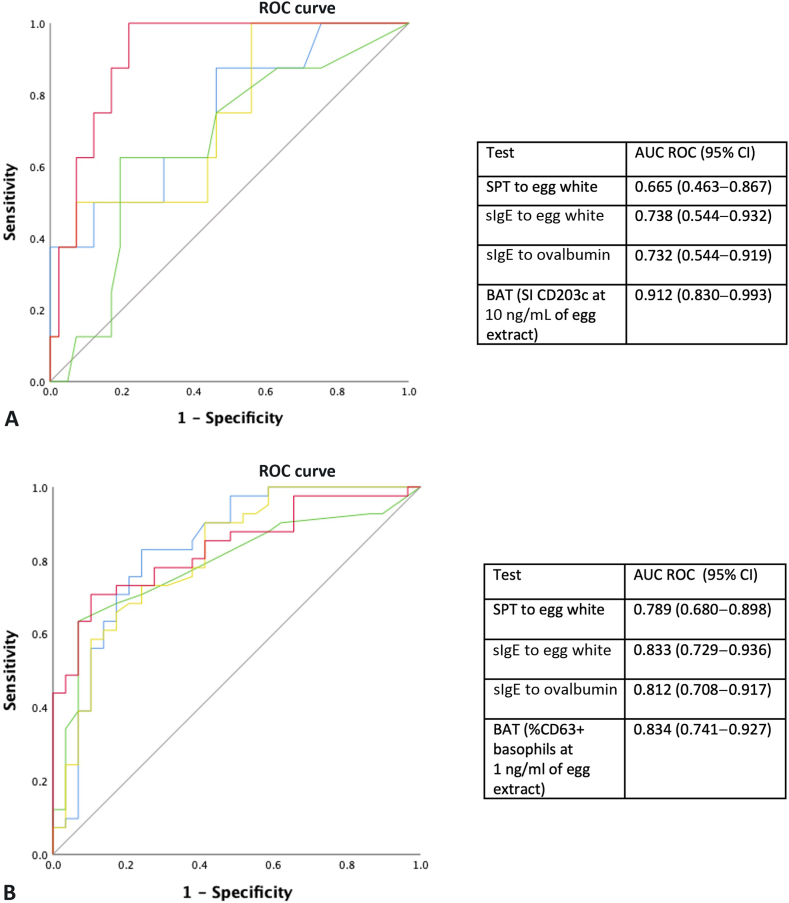
ROC curve for SPT to EW extract, sIgE to EW, sIgE to OVA and the BAT using CD63 and CD203c at different concentrations of EW or BE for (**A**) consumers and (**B**) avoiders.

We recently reported the diagnostic performance of a variety of tests to predict the outcome of DBPCFCs – BAT and sIgE to EW were the best tests overall. As some patients reported consuming egg in the diet when they entered the study for assessment for egg allergy we wanted to see whether egg consumption affected the results of IgE, IgG4, BAT, and SPT and whether it modified the ability of tests to predict OFC outcomes. For consumers, BAT stood out as the best test to identify patients who reacted to BE (Figure 3, *A*); for avoiders, the best tests were BAT and sIgE to EW (Figure 3, *B*), similar to what we observed,^12^ for the whole population. Table I represents the diagnostic cut-offs for the best performer for each test modality in egg consumers and avoiders.

**Table I:** Cut-offs determined using ROC curve analyses in consumers and avoiders: optimal cut-offs defined by the Youden index and 100% sensitivity and 100% specificity cut-offs are indicated

Diagnostic tests	Cut-off		AUC ROC	Sensitivity	Specificity	PPV	NPV	Diagnostic accuracy	TP/FP	TN/FN
Egg consumers										
SPT EW extract (mm)	100% S	0	0.5	100%	0%	14%	N/A	14%	8/50	0/0
	Optimal	3	0.723	63%	82%	36%	93%	79%	5/9	41/3
	100% Sp	20	0.5	0%	100%	N/A	86%	86%	0/0	50/8
sIgE EW (kU/L)	100% S	0.19	0.61	100%	22%	17%	100%	33%	8/39	11/0
	Optimal	0.52	0.698	88%	52%	23%	96%	57%	7/24	26/1
	100% Sp	7.09	0.688	38%	100%	100%	91%	91%	3/0	50/5
sIgE OVA (kU/L)	100% S	0.23	0.710	100%	52%	22%	100%	50%	8/29	21/0
	Optimal	3.15	0.710	50%	92%	50%	92%	86%	4/4	46/4
	100% Sp	8.54	0.563	13%	100%	100%	88%	88%	1/0	50/7
BAT EW (%CD63 100 ng/mL)	100% S									
	Optimal Youden	4.5	0.776	100%	74%	38%	100%	78%	8/13	37/0
	100% Sp	49.2	0.879	13%	100%	100%	88%	88%	1/0	50/7
BAT EW (SI CD203c 10 ng/mL)	100% S									
	Optimal Youden	1.27	0.844	100%	82%	47%	100%	84%	8/9	41/0
	100% Sp	3.65	0.879	13%	100%	100%	88%	88%	1/0	50/7

Diagnostic tests	Cut-off		AUC ROC	Sensitivity	Specificity	PPV	NPV	Diagnostic accuracy	TP/FP	TN/FN
Egg avoiders										
SPT EW extract (mm)	100% S	0	0.5	100%	0%	59%	NA	59%	41/29	0/0
	Optimal	5	0.783	63%	93%	93%	64%	76%	26/2	27/15
	100% Sp	8	0.561	12%	100%	100%	45%	49%	5/0	29/36
sIgE EW (kU/L)	100% S	0.33	0.707	100%	41%	71%	100%	76%	41/17	12/0
	Optimal	1.54	0.794	83%	76%	83%	76%	80%	34/7	22/7
	100% Sp	26.3	0.537	7%	100%	100%	43%	46%	3/0	29/38
sIgE OVA (kU/L)	100% S	0.20	0.707	100%	41%	71%	100%	76%	41/17	12/0
	OPTIMAL	1.29	0.746	73%	76%	81%	67%	74%	30/7	22/11
	100% Sp	21.65	0.537	7%	100%	100%	43%	46%	3/0	29/38
BAT BE (%CD6 31 ng/mL)	100% S	-2.5	0.517	100%	3%	59%	100%	60%	41/28	1/0
	Optimal Youden	2.2	0.786	71%	90%	91%	64%	79%	29/3	26/12
	100% Sp	11.1	0.671	44%	100%	100%	56%	67%	18/0	29/23
BAT EW (%CD63 100 ng/mL)	100% S									
	Optimal Youden	2.2	0.742	100%	48%	73%	100%	79%	41/15	14/0
	100% Sp	48.8	0.586	29%	100%	100%	50%	56%	12/0	29/29

*FN,* False negatives; *FP,* false positives; *NPV,* negative predictive value; *PPV,* positive predictive value; *S,* sensitivity; *Sp,* specificity; *TP,* true positives; *TN,* true negatives.

### Combination of tests for egg consumers and avoiders to ensure optimization of resources and outcome

**Figure 4:**
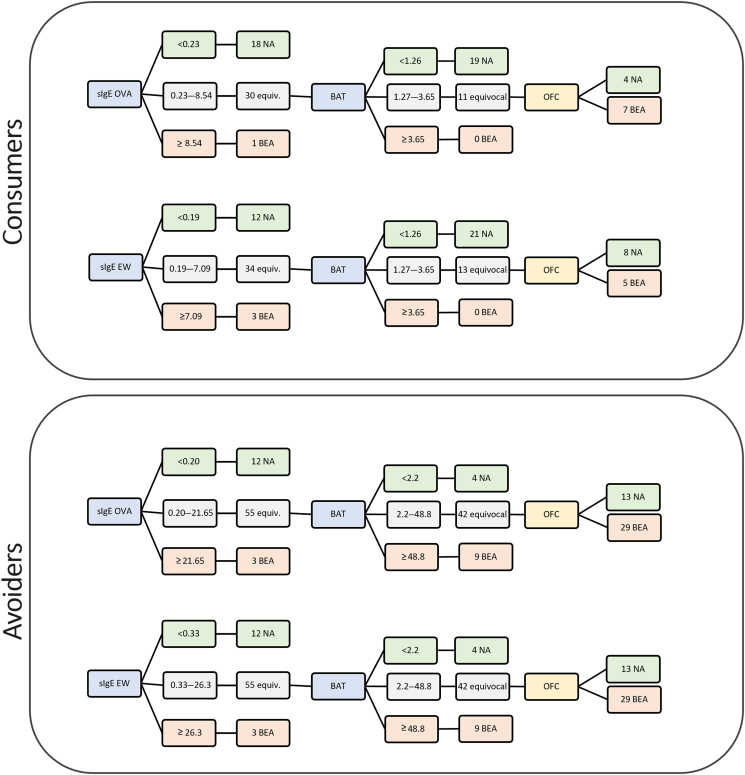
Sequential use of diagnostic tests to predict the outcome of oral food challenges (OFC) to BE with BAT as a second step in the diagnostic process. Positive cut-offs were used to confirm allergy, negative cut-offs to exclude allergy, and patients with results between cut-offs would need an additional test (BAT) or an OFC. *equiv.,* Equivocal; *NA,* not allergic.

When we applied 100% sensitivity and 100% specificity cut-offs for each test, we reached 100% diagnostic accuracy (Figure 4). Compared with the other tests, BAT enabled the lowest number of OFC for both consumers and avoiders, but particularly for consumers (Table II).

**Table II:** Number of OFCs required with individual tests using positive (ie, 100% specificity) and negative (ie, 100% sensitivity) cut-offs. Positive cut-offs were used to confirm allergy, negative cut-offs to exclude allergy, and patients with results between cut-offs would need an OFC - the proportion of cases needing an OFC in relation to all patients (%OFC) and the proportion of positive OFC (%OFC+) are indicated

Egg consumption	Tests	Not allergic	Equivocal	Allergic	OFC–	OFC+	%OFC	%OFC+
Consumers (n = 49)	SPT RE	23	26	0	18	8	26/49 (53%)	8/26 (31%)
	sIgE EW	10	36	3	31	5	36/49 (73%)	5/36 (14%)
	sIgE OVA	18	30	1	23	7	30/49 (61%)	7/30 (23%)
	BAT SI CD203c EW 10	32	16	1	9	7	16/49 (33%)	7/16 (44%)
	sIgE OVA → BAT	37	11	1	4	7	11/49 (22%)	7/11 (64%)
	sIgE EW → BAT	33	13	3	8	5	13/49 (27%)	5/13 (38%)
Avoiders (n = 70)	SPT EW	0	65	5	29	36	65/70 (93%)	36/65 (55%)
	sIgE EW	12	55	3	17	38	55/70 (79%)	38/55 (69%)
	BAT CD63 BE1	1	51	18	28	23	51/70 (73%)	23/51 (45%)
	sIgE OVA → BAT	16	42	12	13	29	42/70 (60%)	29/42 (69%)
	sIgE EW → BAT	16	42	12	13	29	42/70 (60%)	29/42 (69%)

*OFC+,* positive OFC; *OFC–,* negative OFC.

Like previously, we combined tests sequentially to reduce the number of BAT performed keeping with 100% diagnostic accuracy (Figure 4). In consumers, sIgE to OVA followed by BAT required OFC in 22% of patients and 64% of OFC were positive. An sIgE to EW followed by BAT resulted in the need for OFC in 27% of consumers with 38% of OFC being positive. In avoiders, both combinations (ie, sIgE to OVA followed by BAT and sIgE to EW followed by BAT) would require OFC in 60% of patients and 69% of these would be positive. In all these scenarios, BATs were done in 61% to 79% of patients (Table II).

## Discussion

Most egg-allergic children can tolerate some BE^5^; however, it is difficult to precisely estimate how much they are consuming and it can be risky to recommend consumption of BE without doing an OFC to confirm tolerance of an age-appropriate portion of BE.^13^ Given the limited access to OFC, clinical practice is changing toward home introduction of BE in egg-allergic children; however, it is important to ensure the safety of this approach. About 45% of participants in the BAT2 study reported consuming egg in some form and, contrary to what was expected, 38% of these reacted on DBPCFCs to BE. We found that partial egg consumption was associated with immunological changes: avoiders had generally larger SPT, higher levels of sIgE, and higher BAT; and consumers had higher levels of sIgG4. The diagnostic performance of tests differed depending on the egg consumption status with BAT being the best test in consumers and BAT and sIgE to EW being the best tests in avoiders. Using tests sequentially and applying 100% sensitivity and 100% specificity cut-offs before doing the next test allowed for the reduction in the number of tests performed overall. The best sequential combination for consumers was for OVA-sIgE followed by BAT. For avoiders, OVA-sIgE or EW-sIgE followed by BAT led to similar outcomes. This approach required OFC in 22% of consumers and 60% of avoiders and enabled 100% diagnostic accuracy and 100% safety.

The differences in SPT, sIgE, sIgG4, and BAT between egg avoiders and egg consumers suggest immunomodulation by egg consumption; however, the cross-sectional nature of the study design does not allow to establish causality. Overall, consumers had lower SPT results to all forms of egg; lower sIgE levels to egg, to EW, and to OVA (but not to OVM); higher sIgG4 levels to egg, EW, OVA, and OVM; and lower basophil activation to all concentrations of egg extract from 1 ng to 10,000 ng/mL (but not controls), compared with avoiders. Considering, not only egg consumption, but also the allergic status, different tests were affected in different ways. For instance, for SPT, there was no statistically significant difference between allergic avoiders and allergic consumers or between avoiders and consumers who turned out to be tolerant to BE or even between allergic and tolerant among avoiders, suggesting that it is the egg consumption, in the absence of allergy, that reduces skin reactivity and that, even in children who are tolerant, SPT remains high if they are not consuming egg. In terms of sIgE to EW, whose results were similar to sIgE to OVA, there was a significant difference between allergic and tolerant among either avoiders or consumers, more so among consumers, and levels were also higher in allergic consumers compared with allergic avoiders, suggesting that, in allergic patients, egg consumption can boost sIgE production. This was also observed in IgE titers to OVM, in which there was, not only a difference between allergic and tolerant, but also among tolerant, with consumption being associated with higher IgE levels to OVM compared with avoiders. Nevertheless, in respect to egg, EW, and OVA, the sIgE levels between allergic and tolerant among consumers was not significantly different. Focusing on IgE to egg, the statistically significant differences were observed only between allergic and tolerant, both among avoiders and among consumers. Conversely, sIgG4 production seems to be driven both by egg consumption and by the allergic status: levels were higher in consumers, and, among avoiders, were higher in allergic compared with tolerant children. These differences in titers between the different groups applied to IgG4 to egg, EW, OVA, and OVM. Finally, the BAT was significantly different between allergic and tolerant only, either overall, among avoiders, or among consumers. This seems to reflect the relative amount of allergen-sIgE and -sIgG4 in each group of patients: in BE-allergic avoiders the levels of both sIgE-EW and sIgG4-EW were high and they had the highest BAT; the BE-tolerant avoiders had lower levels of sIgE and sIgG4 and lower BAT; and, in the remaining 2 groups, the interplay between IgE and IgG4 may explain the BAT results, which were lowest in BE-tolerant consumers in whom IgG4 was highest and can counteract the effect of IgE (Table E3; available in this article’s Online Repository at www.jaci-inpractice.org).

Previous studies had shown different levels of IgE and different degree of basophil activation in patients with different phenotypes of egg allergy.^6^ From a diagnostic standpoint, reports have been contradictory and have generally showed disappointing prediction power for SPT and allergen-specific IgE.^5^^,^^10^ For instance, patients with large SPT have been reported to pass OFC to BE.^10^^,^^14^ Of course, there are differences in study design with many of previously published studies being retrospective, with relatively low cumulative dose of egg protein reached during BE OFC. sIgE to OVM was shown to be more predictive of the outcome of OFC to BE than sIgE to EW in some but not other studies.^15^^,^^16^ The role of subclinical partial egg consumption and the fact that the various tests reflect different compartments of the immune system, namely cutaneous versus systemic immune responses, may explain the low utility of SPT and sIgE reported in past studies in which egg consumption was not measured. Nevertheless, in a previous retrospective study, BE ingestion was not associated with change in SPT results over time.^14^

In our study, the diagnostic performance of the tests varied when considering the whole population, or when considering both allergic status and egg consumption in the diet. The BAT performed spectacularly better than the other tests in identifying allergic patients among egg consumers, which could markedly reduce the number of OFCs needed and ensure safe introduction of BE in the diet of partial egg consumers with a negative BAT. The performance of BAT and the other tests among avoiders is more similar to their performance in the whole population, considering both consumers and avoiders. The fact that BAT stood out from the other tests in consumers suggests that it could be useful in identifying patients who can consume BE safely at home and the patients who should have an OFC in the hospital to assess whether they can tolerate BE. In avoiders, supervised introduction in OFC may be advisable, similar to what has been suggested in other studies.^13^

The BAT, on its own or used after sIgE testing, enabled the lowest number of OFCs, albeit sometimes with a higher proportion of positive OFCs. This is following the model of 100% diagnostic accuracy, using 100% specificity and 100% sensitivity cut-offs to confirm and rule out BE allergy, respectively, and doing another test or OFC in the equivocal cases. This way, each child seen with suspected BE allergy would have a more precise diagnosis, enabling the prevention of allergic reactions in the community, particularly reactions to foods with higher egg content or reactions in which cofactors could work as facilitators, by reducing the threshold of reactivity and potentially inducing more severe reactions.

The relative smaller number of patients when allergic and tolerant groups are subdivided according to egg consumption is a possible limitation of the analyses we report here. Furthermore, the cross-sectional nature of the BAT2 study does not allow us to confirm the immunomodulatory effect of egg consumption with absolute certainty, which would require an interventional study. However, ethical issues may hamper the performance of such an interventional study because evidence supports early introduction of egg in the diet to prevent egg allergy in infants, and, if children become egg-allergic, consumption of BE improves their nutrition and their quality of life and is associated with better prognosis.^3^, ^4^, ^5^

Whether consumption of BE accelerates resolution of egg allergy remains controversial.^17^^,^^18^ Immunotherapy with EW powder has been shown to be more effective than BE immunotherapy in inducing desensitization, probably owing to its higher immunogenicity.^19^ In any case, consumption of BE is associated with better nutrition and quality of life. Thus, it is important to establish, at the time of diagnosis, whether egg-allergic children can consume BE. Many egg-allergic children avoid egg strictly following their index reaction to egg.^13^^,^^20^ Other children report consuming small amounts of egg but at a dose or frequency that is below the age-appropriate portion; liberalizing the consumption of BE in these cases could lead to accidental allergic reactions, owing to either ingestion of a higher dose than the tolerated one and/or the presence of cofactors, such as viral infection, exercise, menstruation. and nonsteroidal anti-inflammatory drugs, which can reduce the threshold of reactivity. Thus, it can be difficult to advise about safe consumption of BE in egg-allergic children without doing an OFC.^13^

The BAT was shown to be a particularly precise biomarker of allergy versus tolerance in egg-allergic children reporting partial consumption of BE and could be used to identify patients who tolerate BE. Egg allergy is often seen as benign and patients reporting consumption of BE are usually advised to continue. However, in the BAT2 study, 17% of patients consuming egg at study entry reacted on DBPCFCs and 13 patients (22% of positive OFC) required treatment with intra-muscular adrenaline. Therefore, one needs to apply the diagnostic workup cautiously and adjust it to the spectrum of patients seen in a specific clinical context. From our data, it seems that BAT could be used to complement the risk profile of egg-allergic patients, particularly of those reporting egg consumption, before advising whether BE can be consumed, while still avoiding whole egg and RE in the diet.

Our findings show an association between egg consumption and an immunological profile, suggesting that the consumption of egg in the diet can induce immunological changes and modify the diagnostic performance of tests. Although the cross-sectional study design does not allow us to establish causality, our data highlight the importance of documenting egg consumption as part of the clinical history. Documentation of consumption of egg below the amount or frequency defined as age-appropriate dose or strict egg avoidance could allow to segregate patients into 2 different groups, consumers versus avoiders, and their test results could be interpreted differently. The cut-offs indicated here require external validation in an independent population to support its generalizability, which we are in the process of doing as part of the BAT Impact study (NCT05309772). To document egg consumption in the clinic, the role of a specialized dietitian is invaluable, and FFQs can be used to assess this in a more standardized way.

In summary, documenting the frequency, degree of processing, and amounts of egg consumed is extremely important when assessing a child with suspected egg allergy, not only to inform the advice provided in terms of consumption of BE but also to inform the interpretation of allergy test results. The comparison of BAT results across groups reflects the superior accuracy of BAT to distinguish allergy from tolerance, both among avoiders and among consumers. Overall, our study supports the incorporation of BAT in the diagnostic workup of BE allergy to reduce the number of OFCs and improve diagnostic accuracy when assessing whether egg-allergic children can tolerate BE. Using sIgE and BAT sequentially can reduce the number of patients tested on BAT, making the whole process more practical and potentially more cost-effective.

## Figures and Tables

**Table E1:** Dose regimens in grams of protein for baked egg challenges

Age	6–12 mo	1–3 y	4–10 y	11–16 y
Type of challenge	Open	DBPCFC	DBPCFC	DBPCFC
Placebo doses randomly interspersed, n	0	1	1	1
Active doses (g)				
High-risk dose 1∗	0.003	0.003	0.003	0.003
High-risk dose 2∗	0.01	0.01	0.01	0.01
Dose 1	0.03	0.03	0.03	0.03
Dose 2	0.1	0.1	0.1	0.1
Dose 3	0.2	0.3	0.3	0.3
Dose 4	0.4	0.6	0.6	0.6
Open dose	0.8	1.0	1.5	2.5
Cumulative protein dose†	1.53	2.03	2.53	3.53
Total number of muffin/cookies	1.0	1.3	1.6	2.2

*DBPCFC,* Double-blind placebo-controlled food challenge.

∗ The initial doses will be given only in patients considered to be high-risk.

† Cumulative dose does not include the high-risk doses.

**Table E2:** Demographic and clinical characteristics of children being assessed for egg allergy, divided in groups depending on whether they were consuming any egg (consumers) or were avoiding all egg strictly (avoiders).

Demographic and clinical characteristics	BE consumers (n = 62)	BE avoiders (n = 77)	*P* value
Age (y), n (range)	5 (3–8)	5 (2–9)	.852
Sex, n (% females)	24 (39)	35 (46)	.491
Race, n (%)			
White	40 (65)	48 (62)	
Black	3 (5)	6 (8)	.826
Asian	6 (10)	5 (7)	
Chinese	1 (2)	3 (4)	
Mixed	6 (13)	12 (16)	
Other	4 (7)	3 (4)	
Atopic eczema, n (%)	49 (79)	65 (84)	.818
Allergic rhinitis, n (%)	26 (42)	29 (38)	.727
Asthma (%)	15 (24)	27 (35)	.195
SPT to EW extract (mm)	3 (1.5–3.5)	4 (3–6)	<.001∗
SPT to RE (mm)	6 (4; 9)	8 (6–10.5)	<.001∗
SPT to BE slurry (mm)	0 (0;2)	2 (0–3)	<.001∗
Total IgE (kU_A_/L), n (range)	217 (70.5–1,100.8)	219 (58.5–822)	.707
sIgE to egg (kU_A_/L)	0.59 (0.22–2.38)	2.29 (0.64–4.90)	.002∗
sIgE to EW (kU_A_/L)	0.56 (0.21–2.18)	2.29 (0.64–4.90)	.001∗
sIgE to Gal d 1 (kU_A_/L)	0.20 (0.06–0.84)	0.39 (0.02–2.73)	.231
sIgE to Gal d 2 (kU_A_/L)	0.50 (0.17–1.10)	1.29 (0.44–2.68)	.001∗
sIgG4 to egg (mg/L)	0.84 (0.23–3.69)	0.12 (0.05–0.44)	<.001∗
sIgG4 to EW (mg/L)	0.69 (0.18–2.88)	0.09 (0.02–0.36)	<.001∗
sIgG4 to Gal d 1 (mg/L)	0.09 (0.01–1.14)	0.01 (0.01–0.10)	<.001∗
sIgG4 to Gal d 2 (mg/L))	0.59 (0.18–2.77)	0.09 (0.02–0.35)	<.001∗
BAT to EW 100 ng/mL (%CD63+ basophils), n (%)	2.0 (0.3–8.9)	12.9 (2.7–31.9)	<.001∗
BAT with no stimulation (%CD63+ basophils), n (%)	1.9 (1.5; 2.6)	1.8 (1.3; 2.3)	.292
BAT to anti-IgE (%CD63+ basophils), n (%)	29.0 (10.1–50.7)	31.8 (15.1–59.7)	.250
BAT to f-MLP (%CD63+ basophils), n (%)	35.9 (21.5–55.7)	37.3 (22.7–51.0)	.819

*BAT,* Basophil activation test; *BE,* baked egg; *EW,* egg white; *f-MLP, N*-formyl-methionyl-leucyl-phenylalanine *IgE,* immunoglobulin E; *IgG4,* immunoglobulin G4; *RE,* raw egg; *sIgE,* specific immunoglobulin E; *SPT,* skin prick test.

∗ Indicate statistical significance values.

**Table E3:** Relative amounts of IgE and IgG4 levels and basophil activation to EW considering allergic status and consumption of egg in the diet

Patient groups	BAT-EW	IgE-EW	IgG4-EW
BE-allergic			
Avoiders	↑↑↑	↑↑	↑↑
Consumers	↑↑	↑↑↑	↑↑
BE-tolerant			
Avoiders	↑	↑	↑
Consumers	o	↑	↑↑↑
